# A Rare Case of Small Lymphocytic Lymphoma (SLL) Arising After Chronic Myeloid Leukemia (CML) in a Single Patient

**DOI:** 10.7759/cureus.85865

**Published:** 2025-06-12

**Authors:** Lydia George, Tijin Mathew, Benjamin Easow, Krishnamohan R Basarakodu

**Affiliations:** 1 Internal Medicine, Southeast Health Medical Center, Dothan, USA; 2 Hematology and Oncology, Southeast Health Medical Center, Dothan, USA

**Keywords:** bcr-abl1, chronic myeloid leukemia (cml), interleukin 3, simultaneous cml and sll, small lymphocytic lymphoma (sll), tyrosine kinase receptor inhibitors

## Abstract

Chronic myeloid leukemia (CML) and chronic lymphocytic leukemia/small lymphocytic lymphoma (CLL/SLL) are among the most common types of leukemia in adults. However, the sequential development of CLL/SLL following a prior diagnosis of CML in the same individual is exceedingly rare. We present the case of a 65-year-old male initially diagnosed with Philadelphia chromosome-positive CML, who subsequently developed SLL, the tissue-based counterpart of CLL. His CML was initially managed with bosutinib, later switched to dasatinib due to side effects, resulting in both cytogenic and molecular response. Two years later, he was diagnosed with SLL. This report explores the potential molecular mechanisms underlying the coexistence of these distinct hematologic malignancies and highlights important considerations in the selection of appropriate treatment strategies

## Introduction

Chronic myeloid leukemia (CML), the most common myeloproliferative neoplasm, is characterized by the BCR-ABL1 gene fusion, resulting from a translocation between parts of the long arms of chromosomes 9 and 22, occurring during a single bone marrow cell division [1]. This translocation produces the Philadelphia chromosome, which leads to constitutive activation of the BCR-ABL1 tyrosine kinase, driving uncontrolled myeloid proliferation. CML accounts for 15% of adult leukemia cases, with the p210 isoform being the most common alteration, representing 95% of the cases [1]. Chronic lymphocytic leukemia/small lymphocytic leukemia (CLL/SLL), by contrast, is the most common leukemia in adults in the Western world and arises from mature B lymphocytes [2]. CLL accounts for 37% of leukemia cases in adults older than 19 years of age [1]. The occurrence of both CML and CLL/SLL in the same patient is exceedingly rare, with only two reported cases of CLL/SLL [1-7] developing after CML in the medical literature. This case report highlights the molecular mechanisms and clinical significance of recognizing dual malignancies. We present the case of a 65-year-old man who developed SLL two years after being diagnosed with CML. 

## Case presentation

A 65-year-old male with a past medical history of Stage IIA squamous cell lung carcinoma, previously treated with carboplatin and paclitaxel and currently in remission, presented to the oncology clinic in August of 2020 after his primary care physician noted an elevated white cell count during routine testing. Initial complete blood count revealed a leukocytosis of 55,000/µL with a myeloid bulge and left shift (Table 1). Peripheral blood flow cytometry showed 0.5% myeloblasts and an increased number of myeloid lineage cells with a left shift. Fluorescence in situ hybridization (FISH) of peripheral blood detected a variant rearrangement of BCR-ABL1 (22q11.22-q11.23/9q34.11-q34.12) in 98% of analyzed cells, consistent with the Philadelphia chromosome and BCR-ABL1 fusion. Bone marrow biopsy demonstrated granulocytosis with granulocytic left shift, consistent with chronic phase chronic myeloid leukemia (CML), along with associated microcytic hypochromic anemia and thrombocytosis.

**Table 1:** Patient's initial CBC and differential values suggestive of marked leukocytosis with left shift, basophilia, and lymphopenia.

CBC and differential	Reference range	Patient values
White Blood Cells	4.5 - 10.0 x 10-3/uL	55 x 10-3/uL
Red Blood Cells	4.40 - 5.90 x 10-6/uL	4.96 x 10-6/uL
Hemoglobin	13.0 - 18.0 gm/dL	13.0 gm/dL
Hemtatocrit	39.8 - 52.2 %	40.0%
Mean Corpuscular Volume	80.0 - 97.0 fL	80.5 fL
Platelets	150 - 450 x 10-3/uL	463 x 10-3/uL
Neutrophils	51 - 67 %	65%
Lymphocytes	25 - 35 %	5.0%
Monocytes	0-15 %	2.0%
Basophils	0-1 %	3.0%
Bands	3 - 5 %	6.0%
Metamyelocytes	0 - 0 %	8.0%
Myelocytes	0 - 0 %	10.0%
Atypical lymphocytes	0- 1 %	1.0%

The patient was initially started on bosutinib, with a good hematologic response. However, therapy was discontinued after two months due to severe gastrointestinal bleeding and anemia. Anemia was treated with intravenous ferric carboxymaltose. Capsule endoscopy identified active hemorrhage and extensive underlying angiodysplasia in the proximal jejunum. After three months, treatment was then transitioned to daily 100 mg dasatinib after considering the patient's underlying comorbidities. This led to a reduction of white blood count from 21.9 x 103/µL to 8.98 x 103/µL. Quantitative reverse transcription polymerase chain reaction (RT-PCR) for BCR-ABL1 showed a log reduction of 0.324 over the course of three months. Dasatinib was later reduced to 70 mg daily due to gastrointestinal side effects, which subsequently improved, and the patient tolerated the therapy well.

**Figure 1:**
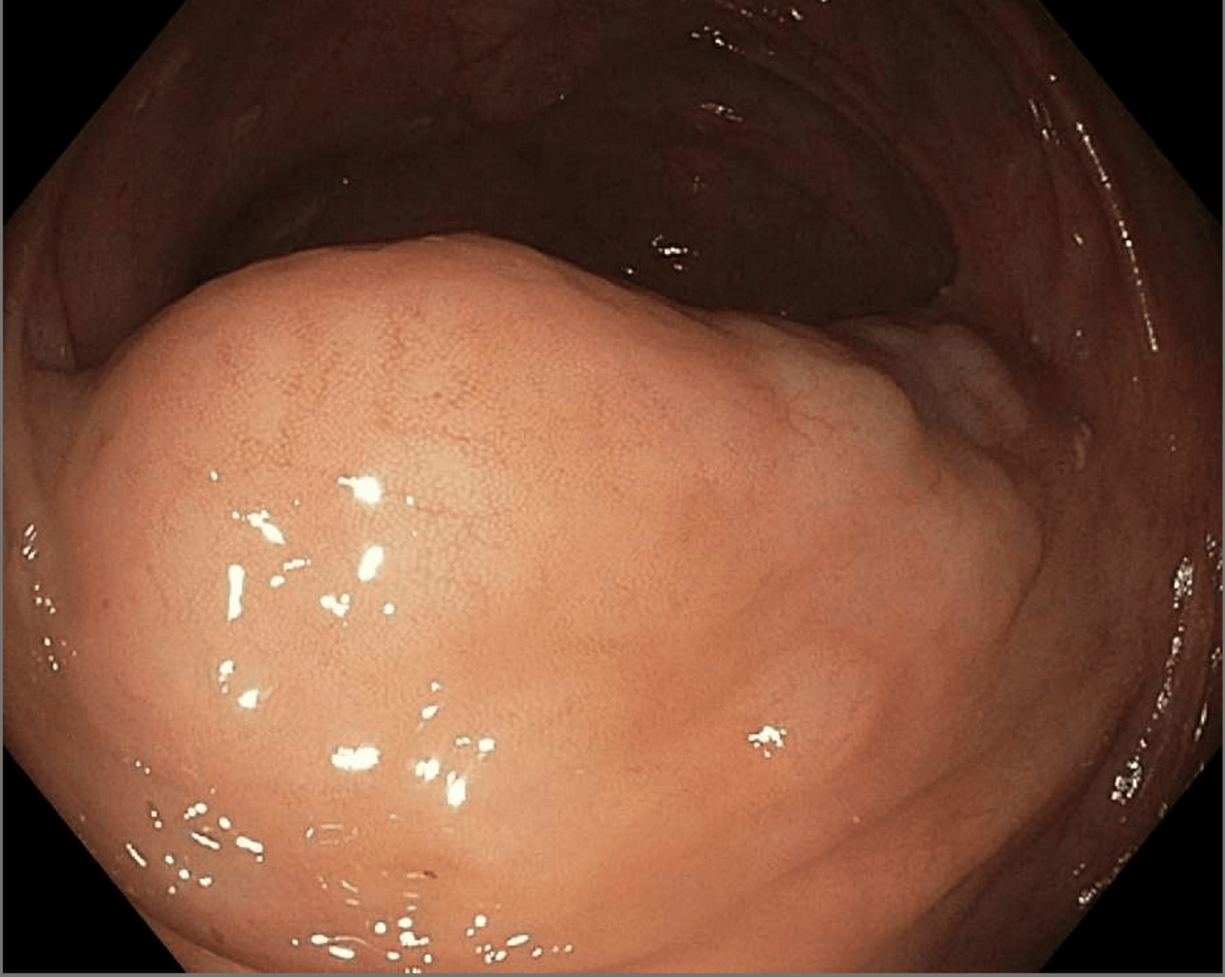
Single 3-cm mass identified in the proximal ascending colon, approximately 2 cm distal to the ileocecal valve.

Two years after initiating dasatinib therapy, a routine surveillance CT scan of the abdomen and pelvis revealed a 2.5 cm cecal mass-like wall thickening accompanied by diffuse, bulky mesenteric lymphadenopathy. Subsequent colonoscopy demonstrated a 3 cm mass in the ascending colon (Figure 1). The lesion lacked the characteristic "pillow sign" typically suggestive of a lipoma. A bite-on-bite biopsy was deferred due to concerns of significant vascularity of the lesion. Given these findings, the patient underwent a robotic-assisted laparoscopic ileocolectomy for definitive diagnosis and management. The cecal mass was successfully excised during the procedure. 

Tissue biopsy results of the resected mass demonstrated a low-grade B-cell lymphoma consistent with small lymphocytic lymphoma (SLL). Immunohistochemistry was consistent with a low Ki-67 proliferation index of 5%, suggesting indolence. Peripheral blood flow cytometry was consistent with 87% monoclonal kappa light chain-restricted B cells expressing CD19, CD20, CD23, CD45, and CD200. Despite an atypical CD5-negative immunophenotype, strong CD23/CD200/LEF-1 expression confirmed SLL diagnosis. The patient continues dasatinib 70 mg daily for CML, and his SLL remains indolent. He is under close clinical surveillance without current need for additional treatment

## Discussion

This case report highlights a rare occurrence of sequential development of chronic myeloid leukemia (CML) followed by small lymphocytic lymphoma (SLL) in a 65-year-old man [1-6]. Although approximately 20 cases of chronic lymphocytic leukemia (CLL) preceding chronic myeloid leukemia (CML) have been documented, only two reported instances describe the subsequent development of small lymphocytic lymphoma (SLL) following a CML diagnosis, with a median interval of 28 months hence highlighting the exceptional rarity of this case [7]. CML is a myeloproliferative disorder characterized by a cytogenic abnormality involving the fusion of the breakpoint cluster region (BCR) gene on long arm of chromosome 9 and ABL proto-oncogene-1 (ABL1) on long arm of chromosome 22, resulting in the characteristic t(9;22) (q34;q11) translocation called the Philadelphia chromosome (Ph). The fusion of these genes causes a constitutive activation of tyrosine kinase, resulting in uncontrolled proliferation of myeloid cells [1].

CML accounts for 15% of adult leukemia cases, with the p210 BCR-ABL isoform being the most common alteration, representing 95% of the cases [2]. In contrast, CLL is a lymphoid malignancy with progressive accumulations of mature monoclonal CD5+ B lymphocytes and accounts for 37% of leukemia cases in adults older than age 19 years, making it the most common leukemia in the USA [1]. Both CLL and SLL can be considered as different manifestations of the same disease, as both share similar histopathological patterns; however, the stark difference lies in the anatomical distribution, CLL primarily in the bone marrow/blood, and SLL in lymph nodes. The occurrence of both CML and SLL could be influenced by interactions between the myeloid and lymphoid cell lineages. A definite mechanism is not yet studied; however, a proposed hypothesis is that the BCR-ABL-transformed cells have been observed to produce cytokines, such as interleukin-3, which enhance the proliferation of B-lymphoid progenitor cells. This mechanism could play a role in the emergence of SLL/CLL in individuals previously diagnosed with CML [3].

Additionally, the development of CLL/SLL in a patient with Ph+ CML most often represents two independent hematologic malignancies. The rarity of Ph+ SLL suggests they likely arose from distinct progenitor clones, unless proven otherwise by cytogenetics. Whether this signifies a defective stem cell microenvironment that triggers leukemogenesis or whether two distinct events occurred by chance in the same individual needs to be further studied [7]. Treatment considerations in such cases are complex. First-line treatment for CML typically involves using a tyrosine kinase inhibitor (TKI) such as imatinib, dasatinib, or bosutinib. In symptomatic patients with markedly elevated leukocyte or platelet counts, short-term cytoreduction with hydroxyurea may be initiated while awaiting confirmatory cytogenic and molecular testing [4]. In contrast, many patients with CLL remain asymptomatic and do not require immediate treatment. For symptomatic or progressive disease, therapeutic options include Bruton tyrosine kinase (BTK) inhibitors (e.g., ibrutinib), BCL-2 inhibitors (e.g., venetoclax), alkylating agents (e.g., chlorambucil), and anti-CD20 monoclonal antibodies (e.g., rituximab) [1].

A major issue arises in treating concurrent CML and CLL/SLL; the concurrent use of both a TKI for CML and a BTK inhibitor may lead to additive toxicity, including cardiotoxicity, neutropenia, thrombocytopenia, and anemia. There are currently no standardized guidelines for the management of concomitant CML and CLL/SLL, emphasizing the clinical need for collaborative registries or multi-center case series to guide future treatment guidelines for patients with concurrent hematological malignancies. Notably, in our patient’s case, SLL remained indolent, and treatment with a TKI such as dasatinib demonstrated a good response. Keeping the side effect profile in mind, modifying the treatment regimen, such as dose reduction or switching to an alternate TKI, is essential in treating such patients.

This case underscores the importance of individualized treatment plans, careful monitoring for disease progression, and adjustment of therapy based on tolerance and clinical course. It also highlights the need for further research to establish evidence-based guidelines for the management of dual hematologic malignancies.

## Conclusions

The development of chronic lymphocytic leukemia/small lymphocytic lymphoma (CLL/SLL) following a diagnosis of chronic myeloid leukemia (CML) is very rare. This case underscores the potential risk for secondary hematologic malignancies in patients with CML, highlighting the importance of maintaining a low threshold for further diagnostic evaluation when new or unexplained symptoms, signs, or lab findings arise. We recommend considering periodic imaging or flow cytometry in long-term tyrosine kinase inhibitor-treated CML patients to facilitate early detection of secondary malignancies. Given the complexity and potential toxicity associated with treating two concurrent hematologic malignancies, we recommend individualized treatment strategies that prioritize the use of a single targeted therapy agent when clinically appropriate. This approach may minimize adverse effects and improve treatment tolerance. In our patient, monotherapy with dasatinib for CML while SLL remained indolent has resulted in a favorable response and good tolerability, supporting its continued use in this context. Ongoing surveillance and further studies are needed to better understand the pathogenesis, optimal management, and long-term outcomes in patients with coexisting CML and CLL/SLL.
